# Alternative splicing variant of the hypoxia marker carbonic anhydrase IX expressed independently of hypoxia and tumour phenotype

**DOI:** 10.1038/sj.bjc.6604111

**Published:** 2007-11-20

**Authors:** M Barathova, M Takacova, T Holotnakova, A Gibadulinova, A Ohradanova, M Zatovicova, A Hulikova, J Kopacek, S Parkkila, C T Supuran, S Pastorekova, J Pastorek

**Affiliations:** 1 Centre of Molecular Medicine, Institute of Virology, Slovak Academy of Sciences, Dubravska cesta 9, Bratislava 845 05, Slovak Republic; 2 Institute of Medical Technology, University of Tampere and Tampere University Hospital, Biokatu 6, Tampere 335 20, Finland; 3 Department of Bioinorganic Chemistry, University of Florence, Polo Scientifico, Via della Lastruccia 3, Sesto Fiorentino 500 19, Italy

**Keywords:** carbonic anhydrase IX, hypoxia, alternative splicing, pH control, carbonic anhydrase inhibitor

## Abstract

CA IX is a hypoxia-induced, cancer-associated carbonic anhydrase isoform with functional involvement in pH control and cell adhesion. Here we describe an alternative splicing variant of the *CA9* mRNA, which does not contain exons 8–9 and is expressed in tumour cells independently of hypoxia. It is also detectable in normal tissues in the absence of the full-length transcript and can therefore produce false-positive data in prognostic studies based on the detection of the hypoxia- and cancer-related *CA9* expression. The splicing variant encodes a truncated CA IX protein lacking the C-terminal part of the catalytic domain. It shows diminished catalytic activity and is intracellular or secreted. When overexpressed, it reduces the capacity of the full-length CA IX protein to acidify extracellular pH of hypoxic cells and to bind carbonic anhydrase inhibitor. HeLa cells transfected with the splicing variant cDNA generate spheroids that do not form compact cores, suggesting that they fail to adapt to hypoxic stress. Our data indicate that the splicing variant can functionally interfere with the full-length CA IX. This might be relevant particularly under conditions of mild hypoxia, when the cells do not suffer from severe acidosis and do not need excessive pH control.

Carbonic anhydrase IX (CA IX) is one of 12 enzymatically active carbonic anhydrase isoforms expressed in the human body. These zinc metalloenzymes catalyse a reversible conversion of carbon dioxide to bicarbonate and proton, and thereby contribute to modulation of ion transport and maintenance of acid-base balance. The CA isoforms show remarkable diversity in their enzyme activity, kinetic properties, distribution in tissues, localisation in subcellular compartments and sensitivity to inhibitors. This diversity enables them to fulfill specific roles in metabolically active organs and in various physiological situations (Pastorekova *et al*, 2004).

In contrast to the majority of CA isoforms that are mostly present in differentiated cells of the normal tissues, CA IX expression is predominantly associated with a broad range of tumours derived from cells, which contain no or low level of CA IX (Potter and Harris, 2003). The only normal tissues that show moderate-to-abundant expression of CA IX belong to gastrointestinal tract and comprise epithelia of the glandular stomach, small intestine, and gallbladder (Pastorekova *et al*, 1997).

The tumour-related expression pattern of CA IX is principally determined by a strong activation of *CA9* gene transcription via a hypoxia-inducible factor (HIF), which binds to hypoxia responsive element (HRE) localised in the minimal *CA9* promoter proximal to transcription start site at −10/−3 position (Wykoff *et al*, 2000). The HIF transcription factor significantly changes the expression profile of weakly oxygenated tumour cells by the activation of genes that either support their survival and adaptation to hypoxic stress or lead to their death. As a result, hypoxia selects more aggressive tumour cells with increased capability to invade and metastasise and is therefore inherently associated with bad prognosis and poor response to anticancer therapy (Harris, 2002).

Strong induction by HIF, hypoxia-related intratumoral distribution, and the relationship to cancer development and/or treatment outcome predispose CA IX to serve as a surrogate marker of hypoxia with a prognostic value (Potter and Harris, 2003). Moreover, CA IX also appears to play an active role in tumour biology via modulation of cell adhesion and control of pH (Svastova *et al*, 2003; Svastova *et al*, 2004; Swietach *et al*, 2007). CA IX participates in bicarbonate transport metabolon and contributes to acidification of extracellular microenvironment in response to hypoxia (Svastova *et al*, 2004; Morgan *et al*, 2007).

Here we show that the *CA9* gene expression involves alternative splicing and describe the alternatively spliced (AS) variant of *CA9* mRNA. We demonstrate that the AS variant is less abundant than the full-length (FL) *CA9* mRNA in tumours, but in contrast, can be detected in normal tissues and under normoxia. The human AS mRNA does not contain exons 8–9 and codes for a truncated CA IX protein. The AS CA IX is not confined to the plasma membrane, shows reduced catalytic activity upon overexpression in HeLa cells, reduces hypoxia-induced extracellular acidification, and compromises growth of HeLa spheroids. Because the AS variant can be present in the normoxic cells with normal phenotype in the absence of FL CA IX, it can produce false-positive results in the studies designed to assess hypoxia- and tumour-related expression of *CA9* gene with prognostic intent. Moreover, it may functionally interfere with the FL CA IX, especially under moderate hypoxia, when the FL levels are relatively low.

## MATERIALS AND METHODS

### Cell culture, tissues, and antibodies

Canine MDCK epithelial cells, human tumour cell lines CAKI-1, and ACHN derived from kidney carcinoma, as well as Caski, SiHa, HeLa, and C33a lines from cervical carcinoma were cultivated in DMEM supplemented with 10% fetal calf serum (FCS) (BioWhittaker, Verviers, Belgium) and 40 *μ*g ml^−1^ gentamicin (Lek, Ljubljana, Slovenia) in a humidified atmosphere with 5% CO_2_ at 37°C. Hypoxic treatments were performed in an anaerobic workstation (Ruskin Technologies, Bridgend, UK) in 2% O_2_, 5% CO_2_, 10% H_2,_ and 83% N_2_ at 37°C. To achieve different cell density, 7 × 10^5^ cells were plated to dishes of different diameters (3.5 cm, 6 cm, and 10 cm), allowed to adhere for 24 h and grow for additional 24 h in normoxia or parallel in hypoxia.

HeLa spheroids were preformed from 400 cells per 20 *μ*l of culture medium in drops hanging on the lid of a tissue culture dish for three days at 37°C. The resulting cell aggregates were transferred to a Petri dish with a non-adherent surface and cultivated in suspension for an additional 11 days, with medium exchange every third day. The spheroids were examined with a Nikon E400 microscope and photographed with a Nikon Coolpix 990 camera.

Human tissues were selected from the collection described previously (Kivela *et al*, 2005). M75 and V/10 mouse MAbs specific for the human MN/CA IX protein were characterised earlier (Pastorekova *et al*, 1992; Zatovicova *et al*, 2003). Rat monoclonal anti-*α*-tubulin antibody (clone YL 1/2) in an undiluted hybridoma medium (described by Kilmartin *et al* 1982, and kindly provided by Professor M Novak, Institute of Neuroimmunology, Slovak Academy of Sciences, Bratislava) was used as a loading control. Secondary anti-mouse and anti-rat peroxidase-conjugated antibodies were from Sevapharma (Prague, Czech Republic). Anti-mouse FITC-conjugated antibodies were from Vector Laboratories (Burlingame, CA, USA). Immunofluorescence was performed as before (Svastova *et al*, 2004). Antibodies against the mouse CA IX protein were described elsewhere (Gut *et al*, 2002).

### Expression plasmids

The eukaryotic expression plasmid pSG5C-AS encoding the human splicing variant was generated by inverse PCR from the pSG5C-MN/*CA9* expression plasmid (Pastorek *et al*, 1994) that contains a full-length human *CA9* cDNA (GenBank no. X66839) using the primers to exons 10 and 7. The forward primer (h10S, 5′-GTGACATCCTAGCCCTGGTTTTT-3′) was specific to the start of exon 10 and the reverse primer (h7A, 5′-CTGCTTAGCACTCAGCATCACTG-3′) was specific to the end of exon 7. The same h7A and h10S primers were used for the preparation of a bacterial expression vector pGEX-3X-AS encoding a GST-fused splice variant of the human CA IX protein, from the primary plasmid construct pGEX-3X-*CA9* coding for the full-length CA IX protein without the signal peptide. PCR amplifications were performed using a Phusion polymerase (Finnzymes, Espoo, Finland). Polymerase chain reactions consisting of an initial denaturing at 98°C for 30 s, 32 cycles of denaturing at 98°C for 10 s, annealing at 64°C for 30 s, extension at 72°C for 1 min 40 s, and final extension for 5 min at 72°C. Polymerase chain reaction products were gel-purified, treated with T4 polynucleotide kinase and ligated with T4 DNA ligase (Invitrogen, Carlsbad, CA, USA). All constructs were verified by sequencing. The construct coding for GST-PGCA fusion protein containing the extracellular part of the human CA IX was described earlier (Zatovicova *et al*, 2003).

### Transfection

The cells were plated in 60 mm Petri dishes to reach approximately 70% density on the next day. Transfection was performed with 2 *μ*g of the pSG5C-AS plasmid together with 200 ng of pSV2neo plasmid using the Gene Porter II transfection reagent (Genlantis, San Diego, CA, USA). The transfected cells were subjected to selection using G418 (Invitrogen, Carlsbad, CA, USA) at a concentration of 900 *μ*g ml^−1^ for HeLa cells, and 500 *μ*g ml^−1^ for MDCK cells. The resistant colonies were cloned, tested for expression of the splicing variant by immunoblotting and expanded.

### Binding of fluorescent CA inhibitor

The fluorescent CA inhibitor (FITC-CAI) was obtained by reaction of homosulphanilamide with fluorescein isothiocyanate and showed a *K*_I_ value of 24 nM towards CA IX (Svastova *et al*, 2004; Cecchi *et al*, 2005). The inhibitor was dissolved in PBS with 20% DMSO at 100 mM concentration and diluted in a culture medium to a final 1 mM concentration just before addition to cells. The MDCK-CA IX cells (Svastova *et al*, 2004) were plated at a density of 400 000 cells per 3.5 cm dish in the medium containing the conditioned medium from MDCK-AS transfectants that secrete the human AS variant. Control cells were incubated in the absence of secreted AS. After 24 h incubation, equivalent fresh media were replenished, FITC-CAI was added to cells, the cells were transferred to the hypoxic workstation, and binding was allowed for additional 48 h. Parallel samples were incubated in normoxia. At the end, the cells were washed five times with PBS and viewed by a Nikon E400 epifluorescence microscope. Intensity of the fluorescence was evaluated from acquired images using the Scion Image *β* 4.02 software (Scion Corporation, Frederick, MD, USA) and relative FITC-CAI binding was expressed in per cent.

### Immunoprecipitation and immunoblotting

Proteins were extracted from the cell monolayer or tissue homogenate with RIPA buffer as described previously (Svastova *et al*, 2004). The samples for the detection of extracellular human AS were prepared from the culture medium of AS-transfected cells incubated without FCS under hypoxia and normoxia for 24 h. One-fourth (500 *μ*l) of the culture medium was 10 times concentrated and separated in SDS–PAGE. For immunoprecipitation, CA IX-specific MAbs in 1 ml of hybridoma medium were bound to 25 *μ*l 50% suspension of Protein-A Sepharose (Pharmacia, Uppsala, Sweden) for 2 h at RT. Cell extract (200 *μ*l) was pre-cleared with 20 *μ*l of 50% suspension of Protein-A Sepharose and then added to the bound MAbs. Immunocomplexes collected on Protein-A Sepharose were washed, boiled, and subjected to SDS–PAGE on 10% gel and to immunoblotting as described previously (Zatovicova *et al*, 2003).

For the isolation of membrane and sub-membrane proteins and analysis of oligomers, the cells were washed with PBS and incubated with RIPA extraction buffer for 30 s on ice. RIPA buffer with proteins was aspirated and fresh RIPA buffer was added to the cells. The remaining proteins were then extracted for 15 min on ice. Oligomers were first immunoprecipitated from HeLa-AS extract using the CA IX-specific MAbs V/10 (recognises FL but not AS) or M75 (recognises both variants). Components of the precipitated oligomers were resolved in reducing SDS–PAGE, blotted and visualised using the peroxidase-labelled M75.

### Reverse transcription PCR

Total RNA was isolated either from cells or from tissues using InstaPure reagent (Eurogentec, Seraing, Belgium). Reverse transcription was performed with M-MuLV reverse transcriptase (Finnzymes, Oy, Finland) using random heptameric primers (400 ng *μ*l^−1^). The mixture of 5 *μ*g of total RNA and random primers (400 ng *μ*l^−1^) was heated for 10 min at 70°C, cooled quickly on ice and supplemented with 0.5 mM dNTPs (Finnzymes), reverse transcriptase buffer containing 6 mM MgCl_2_, 40 mM KCl, 1 mM DTT, 0.1 mg ml^−1^ BSA, and 50 mM Tris–HCl, pH 8.3. The mixture in a final volume of 24 *μ*l was further supplemented with 200 U of reverse transcriptase M-MuLV, incubated for 1 h at 42°C, heated for 15 min at 70°C, and stored at −80°C until used.

PCR was performed with Dynazyme EXT polymerase (Finnzymes) with the primers listed in Supplementary Table 1. The protocol of PCR consisted of 94°C for 3 min followed by 30 cycles of denaturing at 94°C for 30 s, annealing for 40 s (temperature depended on sets of primers), and extension at 72°C for 40 s, followed by a final extension at 72°C for 5 min. Resulting PCR fragments were run on 1.5% agarose gels along with the SmartLadder (Eurogentech, Seraing, Belgium). The intensity of bands corresponding to individual PCR products was evaluated with ImageJ 1.34 s software (Rasband, WS, ImageJ, US National Institutes of Health, Bethesda, MD, USA, http://rsb.info.nih.gov/ij/, 1997–2007). ImageJ can create density histograms and calculate area and pixel value statistics of user-defined selections. The amount of PCR products was semiquantitatively expressed as the ratio of the intensity of each band to the intensity of the related *β*-actin internal standard.

The PCR products were purified and sequenced using an automatic sequencer from Applied Biosystems ABI 3100 (Foster City, CA, USA) to verify their identity.

### Real-time PCR

Total RNA isolated from the human stomach, small intestine, and colon was obtained from BD Biosciences, San Jose, CA, USA. In addition, RNA isolated by Instapure from normoxic and hypoxic SiHa, Caski and ACH carcinoma cells was used. Reverse transcription was performed with M-MuLV reverse transcriptase as described above. Real-time PCR was performed with DyNAmo™ HS SYBR® Green qPCR kit (Finnzymes) with primers specific for FL (h8A) and AS (h10/7A), respectively, combined with h7S primer common for both transcripts. Real-time PCR was run on TECHNE QUANTICA (Duxford, Cambridge, UK), using the following programme, UNG incubation at 50°C for 5 min, initial denaturation at 95°C for 15 min followed by cycling (45 cycles) denaturation at 95°C for 10 s, annealing at 64°C for 20 s, extension at 72°C for 30 s, and final extension for 7 min at 72°C. All PCRs were performed in duplicates and repeated three times. The amount of each type of PCR product was normalised against *β*-actin and the ratio between FL and AS was calculated for each tissue or cell line.

## RESULTS

### Identification and structure of a human splice variant of CA IX

Alternative splicing of *CA9* mRNA was first recognised in our RT–PCR study of the mouse gastrointestinal tissues using the primers for the amplification of exons 6–11. The mouse splicing variant does not contain the exons 7 and 8 (GeneBank no. EF12497) and codes for the CA IX protein with deleted amino acids 335–379, which lacks the C-terminal part of the catalytic (CA) domain (Supplementary Figure 1). The mouse AS protein expressed in the transfected NIH 3T3 and MDCK cells produced a single 48 K band in immunoblotting and exhibited intracellular localisation in immunofluorescence (Supplementary Figure 2).

**Figure 1:**
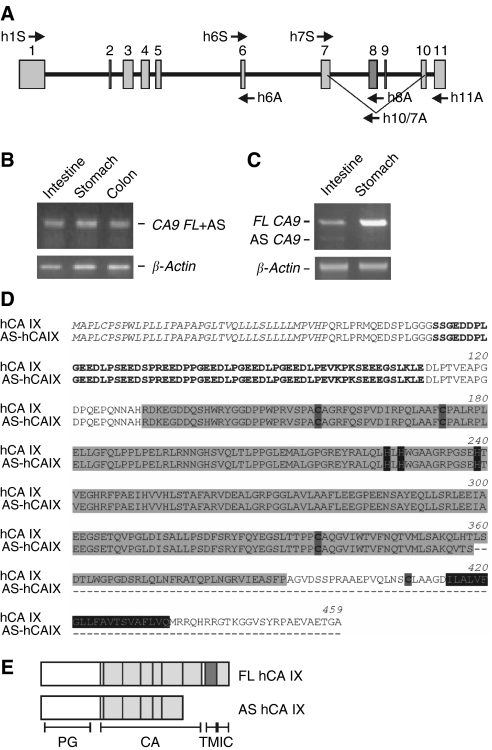
Identification and predicted structure of the human splicing variant of CA IX. (**A**) Schematic illustration of the genomic structure of the human *CA9* gene (GenBank no. Z54349). Positions of primers are indicated by arrows. Exons excluded by alternative splicing are in dark grey colour. (**B**) RT–PCR analysis of *CA9* in the human stomach, small intestine, and colon using h1S-h6A primers that do not discriminate between the splicing variants. (**C**) Amplification of both FL and AS transcripts in the human tissues using h6S-h11A primers. (**D**) Comparison of amino acid sequences deduced from the human FL and AS *CA9* cDNAs. Signal peptide (SP) is written in italic, proteoglycan-like domain (PG) is in bold, carbonic anhydrase domain (CA) is on a grey background, and the transmembrane region (TM) is written by white letters on a black background. Dashed lines represent amino acid residues deleted in AS. Histidines that bind a catalytic zinc and cysteines involved in formation of S-S bonds are placed on a dark background (**E**) Predicted structure of the human FL and AS CA IX proteins.

This finding motivated us to search for the AS *CA9* mRNA in human tissues and cell lines. We designed a set of primers that covered the entire human *CA9* mRNA (Figure 1A). These were employed in RT–PCR on cDNA templates reverse-transcribed from mRNAs isolated from the human stomach and small intestine. Using the primers designed against exons 1 and 6 we detected only a predicted PCR product (Figure 1B). However, the primers to exons 6 and 11 generated two PCR amplicons – a more abundant longer product and a much less abundant shorter product (Figure 1C). The intensity of the FL-related band was 19.8 times higher than that of the AS band in the stomach and 2.6 times in the small intestine. Sequence analysis of the shorter product confirmed that it corresponds to a human AS variant of *CA9* mRNA. The splicing led to a deletion of exons 8 and 9 (GenBank no. EF122496).

To facilitate a separate detection of the FL and AS variants of *CA9* mRNA, we utilised primers that allowed for their individual amplification. The design was based on placing one FL-specific primer (h8A) inside the deleted region and one AS-specific primer (h10/7A) on the alternative splicing-generated junction. Each of these primers was used in combination with h7S primer common for both transcripts (Figure 1A). The resulting fragments were of 154 bp (FL) and 140 bp (AS) in length, respectively. To quantify the amount of FL and AS transcripts and evaluate their relationship, we performed real-time PCR using these primer combinations. Results of three times repeated analyses revealed that the ratio of FL to AS was 19.6±1.2 for the stomach (which is known to express a very high level of FL), 17.3±1.1 for the small intestine, and 5.4±0.1 for the colon (which is known to express lower level of FL than the stomach).

Computer-predicted human AS CA IX protein is lacking the amino acids 356–412 and its deduced molecular weight is about 43 K compared to a predicted size of 49 K for the full-length (FL) CA IX. The deletion eliminated 35 amino acids from the C-terminal part of the catalytic CA domain and 21 amino acids localised between the CA domain and the transmembrane region, which include Cys^409^ that appears to participate in the formation of intermolecular S–S bonds (Figure 1D). Owing to a frameshift-generated stop codon at position 1119 bp in AS mRNA (in *FL CA9* mRNA, the stop codon is at position 1142 bp), the AS protein is truncated and contains neither the transmembrane nor the intracytoplasmic domains (Figure 1E).

### Expression of human AS CA IX in tumour cell lines and tissues

**Figure 2:**
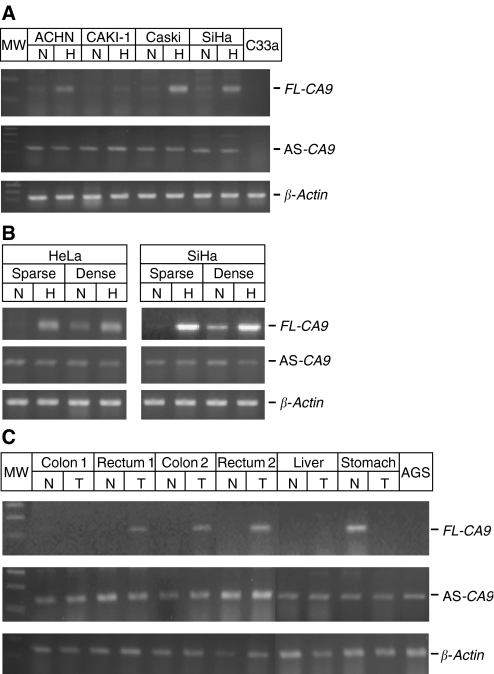
Expression of the AS CA IX variant in human tumour cell lines and in human tissues. RT–PCR analysis of human AS *CA9* using the primers designed for individual amplification of the splicing variants, namely h7S-h8A for FL and h7S-h10/7A for AS (see Figure 3). *β*-actin was used as a standard. SmartLadder was included in the right side of the gels. The cDNAs were isolated (**A**) from the cells exposed to normoxia (N) and hypoxia (H) for 48 h, (**B**) from the cells incubated at low and high density for 72 h, and (**C**) from normal and tumour human tissues. The results indicate that the AS expression is steady and does not depend on hypoxia, density, and tumour phenotype.

The primers suitable for individual amplification of AS and FL were also used for the analysis of tumour cell lines and tissues. First, we analysed the presence of the AS variant in the human cancer cell lines exposed to hypoxia (2%) and normoxia (21%). The AS variant was detected in all examined cell lines and displayed similar levels under both normoxia and hypoxia (Figure 2A). This was in contrast to FL *CA9* mRNA, which was clearly hypoxia-inducible and showed considerably increased levels, namely in Caski, SiHa, and ACHN cells, whereas CAKI-1 cells expressed only a very low level of FL *CA9* (Figure 2A). No FL *CA9*-specific signal was observed in C33a cells that lack the *CA9* gene (Lieskovska *et al*, 1999). Quantitative real-time PCR confirmed these data by showing considerable increase of FL to AS ratio under hypoxic conditions when compared to normoxia (5-fold in SiHa cells, 27-fold in Caski cells, and 8-fold in ACHN cells).

Previous studies have shown a density-induced expression of the FL CA IX that was associated with pericellular hypoxia (Kaluz *et al*, 2002). To see whether the expression of the AS variant is density-dependent, we used HeLa and SiHa cells cultivated in sparse culture (plated at 10 000 cells per cm^2^) and dense culture (80 000 cells per cm^2^), respectively, for 24 h. The dense cells clearly showed normoxic expression of the FL *CA9* mRNA, although its level was lower than in the hypoxic cells. No remarkable differences were observed between the cells cultivated in sparse and dense monolayer with regard to level of the AS variant (Figure 2B).

Finally, we analysed the AS expression in normal *vs* malignant human tissues, including the stomach, colon, rectum, and liver. Reverse transcriptase PCR revealed the presence of the AS variant in all examined tissues (Figure 2C). In accord with the previous studies, FL transcript was found only in the normal stomach and in tumours derived from the colon and rectum (Saarnio *et al*, 1998; Kivela *et al*, 2005).

### Localisation and basic characteristics of the human AS variant of CA IX

**Figure 3:**
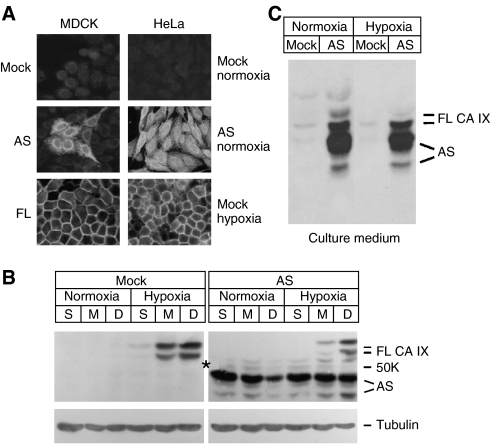
Localisation of the human AS CA IX. CA IX-negative MDCK cells and HeLa cells with natural hypoxia-induced expression of FL CA IX were permanently transfected with AS *CA9* cDNA in pSG5C plasmid. (**A**) Immunofluorescence analysis of the AS-transfected (AS), FL-transfected (FL), and control cells (mock) was performed using M75 MAb recognising both AS and FL proteins. (**B**) Immunoblotting analysis of the protein extracts from HeLa-AS and control HeLa cells plated in sparse (S), medium (M), and dense (D) cultures, allowed to adhere for 24 h and then incubated for 24 h in normoxia and hypoxia, respectively. Asterisk on the CA IX blot indicates the position of 50 K Mw marker, *α*-tubulin was used as a loading control. (**C**) The AS CA IX variant was also detected with M75 MAb in culture medium of AS-transfected cells.

To perform a basic characterisation of the AS variant of CA IX, we generated stable transfectants with ectopic expression of the human AS protein. The human AS cDNA was transfected into CA IX-negative MDCK cells in addition to human HeLa cervical carcinoma cells that naturally express FL CA IX in response to density and hypoxia. In accord with the computer analysis that predicted splicing- and frameshift-mediated removal of TM and IC regions, the AS CA IX protein was not confined to the plasma membrane, but showed intracellular localisation in both, MDCK cells and in normoxic HeLa cells (Figure 3A). This was clearly in contrast with the cell surface localisation of the FL CA IX in the transfected MDCK cells and in the mock-transfected HeLa cells exposed to hypoxia (2% O_2_).

The transfected HeLa-AS cells exhibited in immunoblotting two bands of approximately 43–47 K corresponding to the AS CA IX and additional two bands of 54–58 K corresponding to the hypoxia- and density-induced FL CA IX (Figure 3B). Because of the complete absence of the transmembrane and intracellular domains from the AS protein we also assumed that at least a portion of the AS CA IX molecules should be released into the culture medium. To investigate this possibility, the cells were cultivated under normoxia and hypoxia in the serum-free medium. After 24 h of incubation, one-fourth of the culture medium was concentrated and analysed by SDS–PAGE. Immunoblotting showed the presence of AS CA IX in the culture medium under both, normoxia and hypoxia (Figure 3C). Taken together, these data indicated that the AS is present in the intracellular as well as extracellular space, in contrast to FL CA IX, which is mostly confined to plasma membrane. Moreover, under non-reducing conditions, the FL protein formed oligomers of about 153 K, whereas the AS CA IX variant was unable to do so and was also unable to enter into oligomers built by FL CA IX protein (Supplementary Figure 3, for details see Materials and Methods).

### Functional properties of the human AS CA IX

Expression of the FL CA IX in tumour cells is induced by hypoxia. Hypoxia also activates the catalytic performance of CA IX, which results in enhanced acidification of extracellular pH (Svastova *et al*, 2004). This acidification capacity can be abolished by the overexpression of a dominant-negative mutant lacking the catalytic CA domain of CA IX (Svastova *et al*, 2004). Since the AS protein contains only incomplete CA domain, it was particularly important to analyse whether it is catalytically active and whether it is capable of disturbing acidification mediated by the FL CA IX protein.

Measurement of an enzyme activity was accomplished by stopped flow spectrometry using the recombinant bacterial GST-AS fusion protein containing the truncated CA domain compared to a GST-fused extracellular portion of the FL CA IX containing the complete CA domain (GST-PGCA). The results revealed that the catalytic activity of the wild-type CA IX, *K*_cat_(WT)=3.8 × 10^5^ s^−1^, was reduced to half in the splicing variant, *K*_cat_(AS)=1.9 × 10^5^ s^−1^. In addition, GST-AS protein showed considerably lower affinity for acetazolamine, a sulphonamide inhibitor of carbonic anhydrases: *K*_i_ WT=14 nM *vs K*_i_ AS=110 nM. These data suggest that splicing has compromised both, the enzyme activity of CA IX and its affinity to inhibitors.

**Figure 4:**
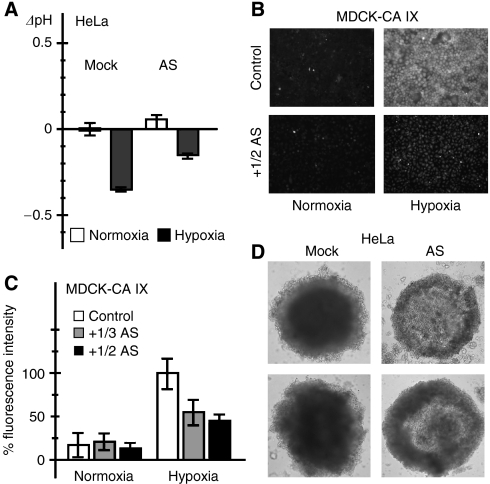
Effect of overexpressed AS variant on acidification, inhibitor binding, and spheroid formation. (**A**) The AS-transfected HeLa cells and related mock-transfected controls were incubated for 48 h in normoxia and hypoxia, respectively, and extracellular pH was measured in culture medium immediately at the end of experiment. Data are expressed as differences between the pH values (ΔpH) measured in normoxic *vs* hypoxic cells and include standard deviations. Results show that expression of AS reduces the acidification mediated by FL CA IX protein under hypoxia. (**B**) MDCK-CA IX transfected cells that constitutively express human FL CA IX protein were treated for 48 h by a fluorescent CA inhibitor (FITC-CAI) in the absence (control) or in the presence of the secreted AS variant added with the conditioned medium from MDCK-AS transfectants. Conditioned medium was mixed with a fresh cultivation medium. FITC-CAI bound only to hypoxic cells and was considerably reduced in the presence of the AS protein. (**C**) The same experiment was performed repeatedly with either one-half (1/2 AS) or one-third (1/3 AS) of conditioned medium from MDCK-AS cells. Binding of FITC-CAI and corresponding fluorescence was evaluated from acquired images using Scion Image software. Data were expressed as a percentage of positive control represented by hypoxic MDCK-CA IX cells incubated with FITC-CAI in the absence of AS. The results confirmed that AS reduces the binding of FITC-CAI to CA IX. (**D**) Microscopic images of spheroids grown from control mock-transfected HeLa cells and from AS-transfected HeLa cells, respectively. Control HeLa cells express hypoxia-induced, functional FL CA IX protein, and produce spheroids that form compact cores. HeLa-AS cells, which contain both hypoxia-induced FL CA IX and constitutively expressed AS, contain loose cores possibly due to AS-compromised function of FL leading to decreased survival of hypoxic core cells.

We also wanted to learn whether the AS CA IX can modulate the capacity of the FL CA IX to acidify extracellular pH under hypoxic conditions. For that purpose we analysed the transfected HeLa-AS cells and the mock-transfected controls incubated for 48 h in 2% O_2_ (hypoxia) and 21% O_2_ (normoxia). Hypoxic incubation led to expected extracellular acidification in the control and in AS-transfected HeLa cells when compared to their normoxic counterparts (Figure 4A). However, the medium was approximately 0.2 pH unit less acidified in the AS-overexpressing cells, suggesting that the AS disturbed the activity of the wild-type CA IX protein.

Since the catalytic site of CA IX is exposed to extracellular space, we tested a possible role of the extracellular fraction of AS. As described earlier, the activity of CA IX can be indirectly demonstrated using the fluorescein-labelled CA inhibitor (FITC-CAI) that binds only to hypoxia-activated CA IX whose catalytic site is accessible by the inhibitor (Svastova *et al*, 2004). Therefore, we used an established model of CA IX-transfected MDCK cells that show CA IX-mediated extracellular acidification when treated by hypoxia and accumulate FITC-CAI in hypoxia but not in normoxia. Here we analysed the accumulation of FITC-CAI in MDCK-CA IX cells in the presence and absence of the culture medium from the AS-secreting MDCK-AS transfectants. As shown on Figure 4B, incubation of MDCK-CA IX cells in the fresh medium mixed with the AS-containing conditioned medium resulted in visibly reduced accumulation of FITC-CA IX supporting the idea that the extracellular AS diminished the binding of the inhibitor. This experiment has been repeated with one-half and one-third of the AS-containing conditioned medium. The acquired images were analysed to determine the differences in the intensity of fluorescence. The results clearly proved that the extracellular fraction of AS reduced FITC-CAI accumulation approximately to one half (Figure 4C).

To see whether the effect of the AS variant on the functioning of the FL CA IX could have biological consequences, we analysed the growth parameters of the HeLa-AS transfectants compared to the mock-transfected controls. No significant differences were observed between these two cell types upon their short-term (72 h) growth in the adherent culture independently of normoxic or hypoxic conditions (data not shown). Therefore, we also produced HeLa cell spheroids grown for 14 days and compared the mass and shape of the spheroids generated from the HeLa-AS cells and the control HeLa cells, respectively. The HeLa-AS spheroids were less compact and lacked the central region, which usually contains the cells that suffer from low oxygen and acidic pH (Figure 4D). The appearance of these HeLa-AS spheroids suggested that the effect of AS, which leads to reduced capacity of the FL CA IX to modulate pH could influence the capability of cells to survive these microenvironmental stresses.

Altogether, our results showed that the AS CA IX is differently regulated, abnormally localised, and functionally diminished when compared to FL CA IX.

## DISCUSSION

Alternative splicing is an important molecular mechanism that significantly contributes to structural and functional diversification of proteins. It frequently results from differential exon inclusion and leads to altered domain composition, subcellular localisation, interaction potential, signalling capacity, and other changes at the protein level. Data obtained by recent genomic technologies indicate that over 60% of human genes are alternatively spliced. It is also becoming more and more evident that imbalances in the expression of alternative splicing variants can significantly affect cell phenotype and play a role in various pathologies (Matlin *et al*, 2005).

Deregulation of alternative splicing is a well-recognised phenomenon particularly in cancer (Venables, 2006). There are numerous examples of alternatively spliced genes whose products are causally involved in tumour progression, such as CD44, HIF-*α*, VEGF, osteopontin, and many others (Gothie *et al*, 2000; Robinson and Stringer, 2001; Wong *et al*, 2003; He *et al*, 2006). In some cases, the splice form that is rare in normal tissues can become common in tumours, while the alternative splice form present in normal tissues can remain constant (Roy *et al*, 2005).

The alternative splicing variant of the human CA IX identified in this study can be classified to this category, although it is difficult to make a clear-cut conclusion, since the expression pattern of the full-length CA IX is quite particular. The FL CA IX is abundant in very few normal tissues including the stomach and small intestine, which at the same time express a low level of the alternative-splicing variant. In gastric carcinomas, the expression of the FL CA IX decreases, but the level of AS is similar as in the normal stomach. In contrast, the expression of the full-length CA IX is absent or very low in the normal colon and rectum (and also in additional normal tissues not analysed in this study) and significantly increases in corresponding tumours (Saarnio *et al*, 1998). However, the AS variant shows a steady expression level in both normal tissues and colorectal carcinomas. These data strongly suggest that its expression is not linked to tumour phenotype. Moreover, in contrast to the FL CA IX whose levels are induced in the cells growing in crowded culture and exposed to low oxygen, the AS variant is not principally dependent on hypoxia and cell density.

Relatively low, but steady expression of AS is of considerable importance for clinical studies using *CA9* transcription as a marker of hypoxic tumours for potential prognostic or predictive purposes. Because of the presence of the AS *CA9* transcript in the normal and/or non-hypoxic tissues, primers or probes designed for detection of the regions that are not affected by splicing cannot differentiate between the two forms of *CA9* mRNA and thus might give false-positive results, which could influence the real clinical value of the hypoxia-induced FL *CA9*.

It is noteworthy that 5′ RACE analysis of the AS mRNA compared to the FL transcript has generated products of identical length supporting the conclusion that both variants are produced from the same promoter (data not shown). This fact might suggest a differential cooperation of the transcriptional apparatus with the components of the splicing machinery in the processing of the *CA9* transcript depending on the physiological circumstances. Indeed, there are several examples of the splicing events regulated by hypoxia such as those related to hTERT, TrkA, and XBP1 (Romero-Ramirez *et al*, 2004; Taconelli *et al*, 2004; Anderson *et al*, 2006). In the case of hTERT, it has been demonstrated that the transcriptional complex containing RNA polymerase II, TFIIB, HIF, and coactivators recruits at the promoter under hypoxia and remains associated with the gene as long as transcription proceeds. This induces a switch in the splice pattern in favour of an active form of the enzyme (Anderson *et al*, 2006). A similar mechanism might operate during the transcription of the *CA9* gene. In addition, recent studies show that splicing can respond to changes in the extracellular microenvironment (Pleiss *et al*, 2007) or to signal transduction through post-translational modifications of spliceosome components (reviewed in Shin and Manley, 2004). For example, activation of the Ras pathway induces inclusion of the metastasis-associated exon v5 in CD44 transcript via phosphorylation of the RNA-binding protein Sam68 and via splicing of the coactivator SRm160 (Matter *et al*, 2002; Cheng and Sharp, 2006). So, it is quite possible that FL splicing is induced in the presence of hypoxia by HIF pathway-stimulated modification of splicing factors.

The AS variant of the human *CA9* mRNA results from the deletion of exons 8 plus 9 and is translated to truncated protein, which does not contain the transmembrane region, intracellular tail, and C-terminal part of the catalytic domain. Removal of the TM and IC regions is apparently responsible for the altered localisation of this AS variant, which predominantly occupies intracellular space and is also released to the extracellular medium. This is in contrast with the FL CA IX protein, which is an integral plasma membrane protein. Such inappropriate localisation linked with a partial deletion of the catalytic domain can be expected to compromise the protein functionality. Indeed, GST-AS shows only one half of the enzyme activity of the corresponding GST-PG+CA protein containing the complete catalytic domain. However, it is very difficult to translate this finding directly into local cellular context, where CA IX interacts with bicarbonate transporters and contributes to pH regulation across the plasma membrane under hypoxic conditions (Svastova *et al*, 2004; Morgan *et al*, 2007; Swietach *et al*, 2007). First, activity measurements were performed with the proteins produced in bacteria in a setting free of any subcellular structures, protein–protein interactions, ion fluxes, and microenvironmental influences, which certainly play a role in modulating the catalytic performance of CA IX. Second, the catalytic activities of different carbonic anhydrase isoenzymes vary roughly within two orders of magnitude, with the highly active isoforms showing from 20 to only 3 times higher activity than the isoenzymes that are considered moderate (Pastorekova *et al*, 2004). So it is not possible to preclude whether the half-reduced activity would be sufficient for the physiological function of CA IX. Anyhow, this question is probably not critical, since the AS variant is not properly localised at the plasma membrane and is unable to form oligomers, which are very important constraints for the CA IX protein functioning.

However, reduced extracellular acidification observed in the culture of hypoxic HeLa-AS cells that constitutively overexpress the AS form of CA IX clearly indicates that it interferes with the function of endogenous, hypoxia-induced FL protein. Although the mechanism is not clear at present, based on the decreased accumulation of CA inhibitor in the hypoxic MDCK-CA IX cells treated with the secreted AS variant, we can propose that AS might compete with the FL CA IX for an interaction with the cell surface components of the bicarbonate transport metabolon (since this interaction appears to include the extracellular portion of CA IX as demonstrated by Morgan *et al*, 2007). Moreover, overexpression of AS considerably affects the capacity of HeLa cells to form compact spheroids, which are often used as a 3D model that mimics tumour mass with corresponding intratumoral microenvironment. Many studies well document gradients of oxygen partial pressure, pH, nutrients, and metabolites across the spheroids whose core regions show a clear analogy with the hypoxic areas of solid tumours that are characterised by a more acidic microenvironment (Alvarez-Pérez *et al*, 2005). It has been shown elsewhere that the plasma membrane staining of the FL CA IX is significantly increased in the innermost cells of multicellular spheroids generated from SiHa and HeLa cervical carcinoma cells (Olive *et al*, 2001; Chrastina *et al*, 2003). These data indicate that FL CA IX is present exactly in the areas where the cells need increased protection and/or adaptation to harmful effects of the hypoxic stress and acidic microenvironment to survive. The FL CA IX acts here via bicarbonate-mediated buffering of intracellular pH (Swietach *et al*, 2007). The AS variant that partially perturbs this pH regulation, obviously does not permit the adaptation to acidic intra-spheroid pH, leading to elimination of the most stressed central cells from the core of spheroids. This idea is consistent with the findings that the catalytic activity of CA IX is regulated by hypoxia and suggests that the capacity of CA IX to modulate pH is vital for the survival of hypoxic tumour cells. The latter suggestion has been indirectly supported also by RNAi experiments by Robertson *et al* (2004). However, it is also possible that the AS protein released to the culture medium can modulate spheroid formation via binding to the transport unrelated receptor and stimulate or block signal transduction in autocrine or paracrine fashion. The literature contains several examples of splicing-truncated proteins functioning in this manner (Aasheim *et al*, 2000; Huang *et al*, 2006).

Although the naturally produced AS variant is expressed at a low level, there are physiological situations and cell types that only weakly induce FL CA IX. For example tumour cells localised at shorter distances from functional blood-supplying vessels are exposed to mild hypoxia and may express comparable levels of FL and AS thus allowing for dominant-negative down modulation of CA IX activity. Such weakly hypoxic cells are presumably not exposed to severe acidosis and therefore may not benefit from full performance of this pH control mechanism. A similar explanation can be applied also to normal tissues suffering from mild ischaemia. This idea finds support in the recent as well as previous data showing that some tumour cell lines, dense normoxic cells (affected by weak pericellular hypoxia) and some early stage less-hypoxic tumours express just low levels of FL CA IX. In conclusion, we propose that the AS variant functions as a modulator of the FL CA IX under circumstances when both proteins are coexpressed.
