# Supplementary figures and images for: Alternative splicing variant of the hypoxia marker carbonic anhydrase IX expressed independently of hypoxia and tumour phenotype
Source: Br J Cancer. 2007 Nov 20;98(1):129–36. doi: 10.1038/sj.bjc.6604111 (PMC2359689; doi:10.1038/sj.bjc.6604111)

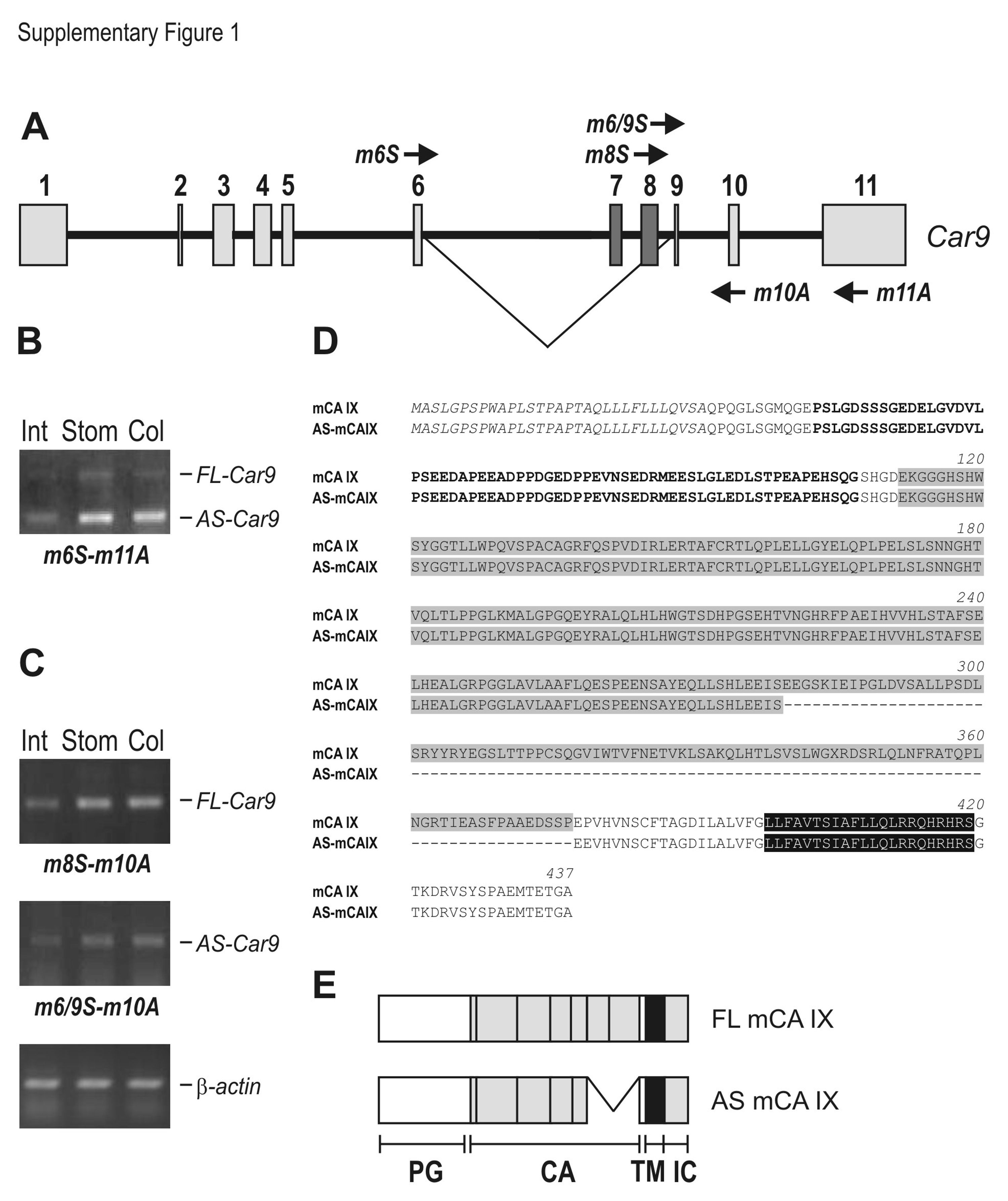

Supplement: Supplementary Figure 1 [file 6604111x1.tif]

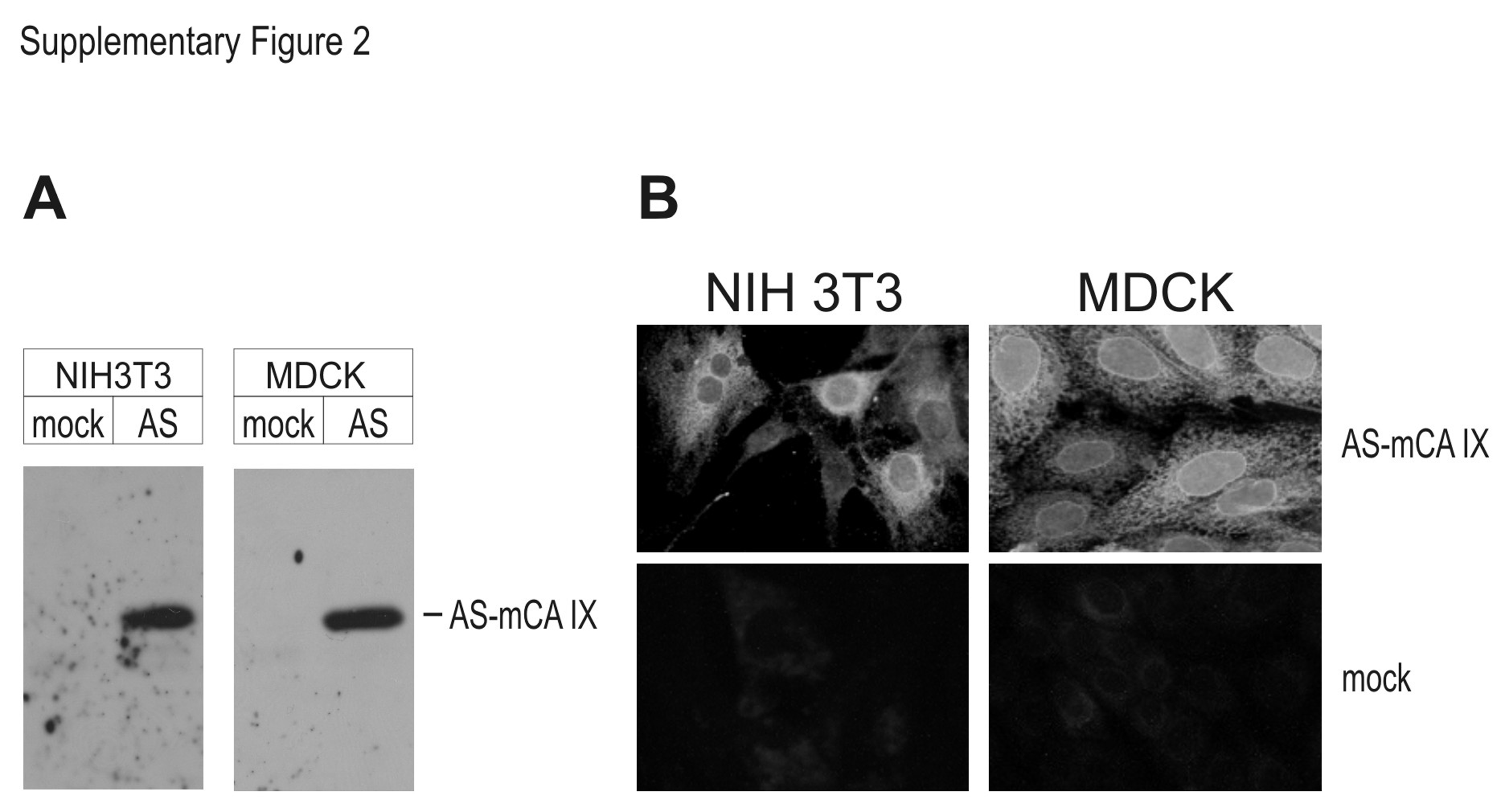

Supplement: Supplementary Figure 2 [file 6604111x2.tif]

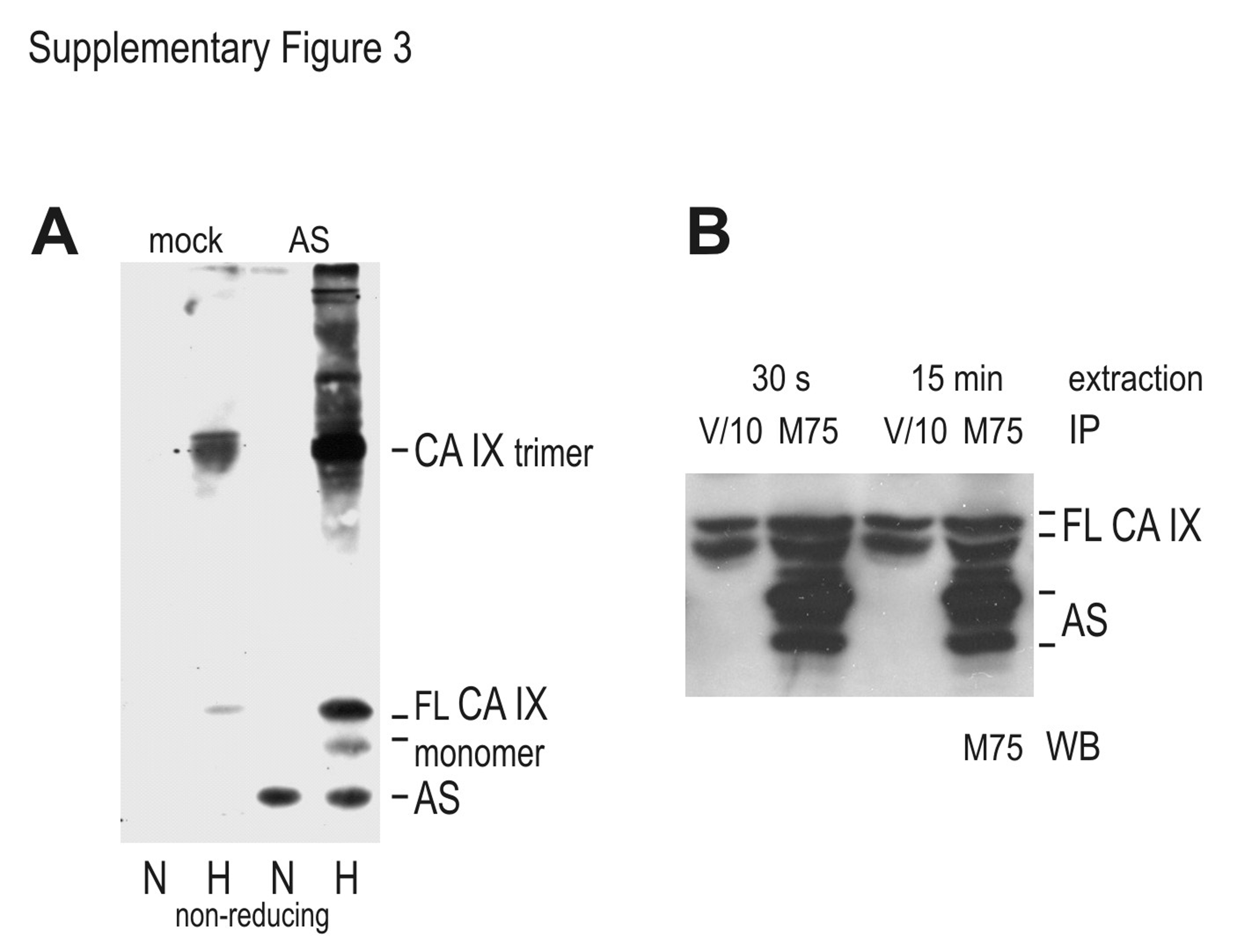

Supplement: Supplementary Figure 3 [file 6604111x3.tif]
