# Supplementary material for: Alternative splicing variant of the hypoxia marker carbonic anhydrase IX expressed independently of hypoxia and tumour phenotype
Source: Br J Cancer. 2007 Nov 20;98(1):129–36. doi: 10.1038/sj.bjc.6604111 (PMC2359689; doi:10.1038/sj.bjc.6604111)
Supplement: Supplementary Figure Legends [file 6604111x4.doc]

**Supplementary Figure 1**

(A) Genomic structure of the mouse *Car9* gene (GenBank # AY049077). (B) RT-PCR of *Car9* splicing variants in the mouse gastrointestinal tissues. (C) Separate amplification of the FL and AS transcripts. (D) Comparison of the FL and AS amino-acid sequences. (E) Predicted structure of the mouse AS CA IX protein.

Supplementary Figure 2

pSG5C-AS plasmid containing the mouse AS cDNA was transfected to NIH3T3 and MDCK cells, respectively. (A) Immunoblotting analysis of AS-transfected cells using the polyclonal serum against the mouse CA IX shows a single AS-related band. (B) Immunofluorescence analysis of the transfectants demonstrates an intracellular localization of the mouse AS protein.

Supplementary Figure 3

(A) Non-reducing SDS-PAGE and immunoblotting with M75 showed that AS is unable to form oligomers. (D) Detection of splicing variants in oligomers by immunoprecipitation from HeLa-AS extract with MAb V/10 (recognizes FL but not AS) or M75 (recognizes both variants). Components of the precipitated oligomers were visualized using peroxidase-labelled M75.

Supplementary Table 1

List of the primers used for PCR amplification of cDNAs encoding the CA9 splicing variants and β-actin internal standards.
