# Supplementary material for: Alternative splicing variant of the hypoxia marker carbonic anhydrase IX expressed independently of hypoxia and tumour phenotype
Source: Br J Cancer. 2007 Nov 20;98(1):129–36. doi: 10.1038/sj.bjc.6604111 (PMC2359689; doi:10.1038/sj.bjc.6604111)
Supplement: Supplementary Table 1 [file 6604111x5.doc]

Supplementary Table 1

| **Primer  designation** | **Position** | **Sequence (5’- 3’)** |
| --- | --- | --- |
| ***mβ−actin S*** | 768-787 | GTTGGCATAGAGGTCTTTACG |
| ***mβ−actin A*** | 968-948 | GCCGCATCCTCTTCCTCCCT |
| ***M6S*** | 794–814 | GGAGGCCTGGCAGTTTTGGCT |
| ***M11A*** | 1358–1336 | CTCCAGTTTCTGTCATCTCTGCC |
| ***M8S*** | 1156-1175 | CCCTGCTGCAGAGGATAGCA |
| ***M10A*** | 1312-1293 | GGTCCCACTTCTGTGCCTGT |
| ***M6/9S*** | 883-893 / 1188-1194 | CTCGGAGGAAG / TCCATGTGAA |
| ***M10A*** | 1312-1293 | GGTCCCACTTCTGTGCCTGT |
| ***hβ−actin S*** | 414-433 | CCAACCGCGGGAAGATGACC |
| ***hβ−actin A*** | 649-629 | GATCTTCATGAGGTAGTCAGT |
| ***h1S*** | 412-433 | GAACCCCAGAATAATGCCCACA |
| ***h6A*** | 924-945 | TCGCTTGGAAGAAATCGCTGAG |
| ***h6S*** | 915-937 | GTTGCTGTCTCGCTTGGAAGAAA |
| ***h11A*** | 1392-1372 | GCGGTAGCTCACACCCCCTTT |
| ***h7S*** | 980-1001 | TATCTGCACTCCTGCCCTCTG |
| ***h8A*** | 1133-1155 | CACAGGGTGTCAGAGAGGGTGT |
| ***h10/7A*** | 1291-1279 / 1106-1095 | CTAGGATGTCAC / CTGCTTAGCACTC |
